# Patients With Berardinelli–Seip and Silver–Russell Syndromes Hospitalized due to Coronavirus Disease (COVID)‐19 in Brazil: A Four‐Year Case Report Profile

**DOI:** 10.1155/crdi/5881408

**Published:** 2026-06-17

**Authors:** Luiz Felipe Azevedo Marques, Adriele Evelyn Ferreira Silva, Patrícia Teixeira Costa, Lucas Silva Mello, Vinícius Santiago dos Santos, Fernando Augusto Lima Marson

**Affiliations:** ^1^ Laboratory of Molecular Biology and Genetics, Postgraduate Program in Health Sciences, Postgraduate Program in Health Data Science, São Francisco University (USF, of the Portuguese Universidade São Francisco), Bragança Paulista, São Paulo, Brazil; ^2^ Laboratory of Clinical and Molecular Microbiology, Postgraduate Program in Health Sciences, Postgraduate Program in Health Data Science, São Francisco University (USF, of the Portuguese Universidade São Francisco), Bragança Paulista, São Paulo, Brazil; ^3^ LunGuardian Research Group, Epidemiology of Respiratory and Infectious Diseases, Postgraduate Program in Health Sciences, Postgraduate Program in Health Data Science, São Francisco University (USF, of the Portuguese Universidade São Francisco), Bragança Paulista, São Paulo, Brazil

**Keywords:** Berardinelli–Seip syndrome, clinical outcomes, COVID-19, hospitalization, rare genetic disorders, SARS-CoV-2, Silver–Russell syndrome

## Abstract

Berardinelli–Seip syndrome is a rare genetic disorder characterized by the near absence of subcutaneous fat and a high prevalence of metabolic complications, such as diabetes mellitus and cardiovascular disease, which may worsen coronavirus disease (COVID)‐19 outcomes. Silver–Russell syndrome, also a rare genetic condition, involves intrauterine growth restriction and multiple metabolic and physical abnormalities that could potentially worsen prognosis after severe acute respiratory syndrome coronavirus 2 (SARS‐CoV‐2) infection. This study analyzed five hospitalized patients in Brazil, two with Berardinelli–Seip syndrome and three with Silver–Russell syndrome, with confirmed COVID‐19. The results showed that most patients presented respiratory symptoms and peripheral oxygen saturation below 95% at admission. Among the cases, two required intensive care and two required noninvasive ventilatory support. Four patients were discharged, while one 19‐year‐old patient with Berardinelli–Seip syndrome, who had multiple comorbidities and was unvaccinated, died. Symptom onset preceded hospitalization by 2–8 days in Berardinelli–Seip and 2–7 days in Silver–Russell patients. Time from symptom onset to outcome ranged from 6 to 10 days. Hospital stays were short (2–6 days). No Berardinelli–Seip patient required intensive care unit admission; two Silver–Russell patients required brief intensive care unit stays. Overall progression remained under 2 weeks. The study did not identify a consistent association between COVID‐19 severity and the presence of these two syndromes, although regular multidisciplinary follow‐up may have contributed to favorable outcomes in patients with Silver–Russell syndrome. Despite diagnostic and data‐source limitations, the findings suggest that these rare conditions were not uniformly associated with poor outcomes, contrary to initial expectations. Further studies with larger cohorts are needed to clarify the interaction between rare diseases and COVID‐19, improve risk stratification, and support clinical management in future public health emergencies.

## 1. Introduction

On March 11, 2020, the World Health Organization (WHO) declared the coronavirus disease (COVID)‐19 a pandemic [1]. Although all people were vulnerable to the virus, individuals with comorbidities were associated with worse outcomes [2–5], especially those with cardiovascular disease, diabetes mellitus, kidney disease, obesity, among others [2, 6]. In view of this, studies have been carried out in an attempt to understand how the pandemic has affected people with comorbidities [7–10]. In this context, the importance of studies on rare diseases in the face of COVID‐19 is highlighted [11, 12]. In contrast to the large number of studies that have analyzed the relationship between comorbidities and COVID‐19 outcomes, there are few studies that have related it to rare diseases [13]. Among the various rare diseases, there are two in which the relationship with COVID‐19 is practically unknown: Berardinelli–Seip syndrome and Silver–Russell syndrome.

Berardinelli–Seip syndrome is a disorder involving adipose tissue—also known as congenital generalized lipodystrophy—an autosomal recessive disease resulting from pathogenic variants in 4 genes: 1‐Acylglycerol‐3‐Phosphate O‐Acyltransferase 2 (*AGPAT2*), Berardinelli–Seip Congenital Lipodystrophy Type 2 (*BSCL2*), Caveolin‐1 (*CAV1*), and Caveolae Associated Protein 1 (*CAVIN1*) and characterized by a shortage of subcutaneous fat [14, 15]. Although extremely rare, with a prevalence of one case per 12 million people [14], of 500 cases described worldwide, 100 are present in Brazil [16]. The clinical features that may indicate a worse prognosis in individuals infected with severe acute respiratory syndrome coronavirus 2 (SARS‐CoV‐2) include hypertriglyceridemia, insulin resistance, diabetes mellitus, cardiovascular disease, hepatomegaly, and acanthosis nigricans [14–16].

The second syndrome, called Silver–Russell, is a rare autosomal dominant disease with at least 16 described genetic pathogenic variants, mainly related to maternal uniparental disomy of chromosome 7 (mUPD7) and methylation abnormalities in chromosome region 11p15, which contains, among others, the Imprinted Maternally Expressed Transcript (*H19*) and Insulin‐Like Growth Factor II (*IGF2*) genes—crucial for intrauterine growth. The syndrome is characterized by intrauterine growth restriction, with clinical findings ranging from growth abnormalities, bone defects, body asymmetry, and hypotrophic muscle mass to heart defects [17]. Its prevalence is described as one case in every 16,000 people [18], and even though modern genetic tests are now available for diagnosis, physical examination is essential due to the remarkable characteristics of the disease [17].

Both syndromes have no cure, and symptom control is the main medical support measure [17]. Their prognosis is diminished by the various metabolic and growth disorders described. Given this and knowing how the pandemic caused by SARS‐CoV‐2 has impacted people with underlying diseases, the aim of this article was to evaluate the clinical impact and outcomes of patients with Berardinelli–Seip and Silver–Russell syndromes hospitalized for COVID‐19 during the four years of the pandemic in Brazil. The data used was collected from a public access platform of the Brazilian Ministry of Health, Open Data of the Brazilian Unified Health System (SUS, of the Portuguese Sistema Único de Saúde) (Open‐Data‐SUS), which gathers data on patients hospitalized for severe acute respiratory infection.

## 2. Case Presentation

The design of the study protocol, as well as the planning of its presentation and manuscript preparation, was based on prior research conducted by our group [3, 4, 19–24]. This study was designed as a descriptive, observational case series based on secondary data extracted from the Brazilian Ministry of Health’s official open‐access platform, Open‐Data‐SUS, available at https://opendatasus.saude.gov.br. Open‐Data‐SUS is a national repository that provides anonymized data from the SUS, supporting epidemiological surveillance, transparency, and public health research.

We accessed the national database of hospitalized cases of severe acute respiratory infection and identified patients hospitalized between 2020 and 2024 with laboratory‐confirmed SARS‐CoV‐2 infection and a recorded diagnosis of Berardinelli–Seip syndrome or Silver–Russell syndrome. Only hospitalized cases with defined outcomes (discharge or death) were included.

The diagnosis of COVID‐19 in all included patients was confirmed by reverse transcription polymerase chain reaction (RT‐PCR) testing for SARS‐CoV‐2. According to the Brazilian Ministry of Health surveillance protocol, RT‐PCR assays were performed on respiratory specimens (nasopharyngeal and/or oropharyngeal swabs) collected during the acute phase of infection.

RT‐PCR is considered the gold standard diagnostic method for COVID‐19 due to its high sensitivity and specificity [25]. The technique involves the extraction of viral ribonucleic acid (RNA) from clinical samples, followed by reverse transcription into complementary deoxyribonucleic acid (cDNA), and subsequent amplification of specific genomic targets of SARS‐CoV‐2. Detection of viral genetic material confirms active infection.

In the Open‐Data‐SUS database, laboratory confirmation is recorded according to standardized national reporting criteria. All cases included in this study were classified as laboratory‐confirmed COVID‐19. Rapid antigen tests or clinical‐epidemiological criteria alone were not used for case confirmation in the analyzed patients.

In accordance with CAse REport (CARE) guidelines for case reports, we extracted all available clinical and demographic variables from the database to provide the broadest possible patient characterization. However, it is important to emphasize that Open‐Data‐SUS is an epidemiological surveillance database designed for monitoring hospitalizations due to COVID‐19 and does not include detailed individualized clinical records. Therefore, information such as comprehensive past medical history, physical examination findings (e.g., respiratory rate, blood pressure, and body mass index), imaging studies (e.g., chest X‐rays or computed tomography), detailed laboratory parameters (e.g., lipid profile, triglycerides, and complete blood chemistry), specific treatment regimens, and individualized therapeutic plans are not available in this dataset. The absence of these variables reflects intrinsic database limitations rather than an omission in data collection by the authors.

The following domains were analyzed:

i. Sociodemographic variables: year and state of notification; sex (male or female); age (years); race/color (White, Black, Mixed, Asian, or Indigenous); area of residence (urban or rural); and COVID‐19 vaccination status.
ii. Clinical presentation: presence of nosocomial infection; signs and symptoms recorded at hospital admission (fever, cough, sore throat, dyspnea, respiratory discomfort, peripheral oxygen saturation < 95%, diarrhea, vomiting, fatigue, and other reported symptoms).
iii. Comorbidities: presence of pre‐existing conditions registered in the system, including cardiovascular disease, diabetes mellitus, hematological disorders, Down syndrome, liver disease, asthma, neurological disorders, chronic lung disease, immunosuppression, kidney disease, obesity, and other reported comorbidities. Only comorbidities explicitly recorded in the database were considered.
iv. Clinical course and outcomes: use of antiviral medication; admission to an intensive care unit; need for ventilatory support (none, noninvasive, or invasive); hospital outcome (discharge or death); time interval between symptom onset and hospitalization; time interval between symptom onset and outcome; time interval between hospitalization and outcome; and length of intensive care unit stay.

Given the small number of identified cases with Berardinelli–Seip and Silver–Russell syndromes, the analysis was purely descriptive. Results are presented as absolute (*N*) values and organized according to the predefined domains. Inferential comparisons with the remaining hospitalized population were not performed due to the limited sample size.

The study was conducted using fully anonymized, publicly available secondary data and followed national ethical regulations and international research principles. In this context, the study was conducted in accordance with the Declaration of Helsinki and approved by the Institutional Research Ethics Committee (Certificate of Presentation for Ethical Appreciation no. 67241323.0.0000.5514; Approval no. 5908611). No written consent has been obtained from the patients as there is no patient‐identifiable data included in this case report.

**FIGURE 1:**
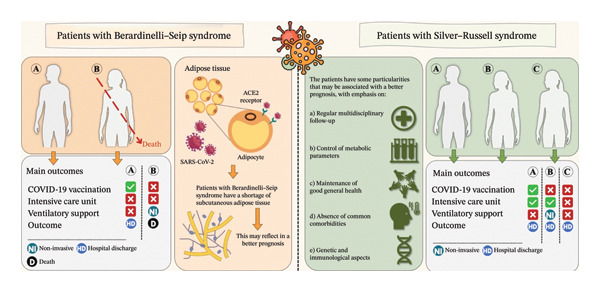
Characteristics of patients with Berardinelli–Seip and Silver–Russell syndromes hospitalized due to coronavirus disease (COVID)‐19 in Brazil. ACE‐2: angiotensin‐converting enzyme 2. SARS‐CoV‐2 (severe acute respiratory syndrome coronavirus 2).

A total of five patients were included in the study, two with Berardinelli–Seip syndrome and three with Silver–Russell syndrome. The patients with congenital generalized lipodystrophy were 11 and 19 years old, male and female, respectively, and both lived in rural areas. On hospital admission, the main symptoms reported were of respiratory origin, including the presence of dyspnea and peripheral oxygen saturation < 95%. No patient was hospitalized in an intensive care unit. Only the 19‐year‐old patient required noninvasive mechanical ventilation; this patient, who was not vaccinated against COVID‐19 and had cardiac, renal, and neurological disorders, died during hospitalization (Table 1, Figure 1).

**TABLE 1:** Clinical and demographic characteristics of patients with Berardinelli–Seip and Silver–Russell syndromes hospitalized due to coronavirus disease (COVID)‐19 in Brazil.

Characteristics	Berardinelli–Seip syndrome		Silver–Russell syndrome		
	Case one	Case two	Case one	Case two	Case three
Notification date	21‐Jul‐22	26‐Mar‐21	21‐Mar‐22	22‐Jan‐22	11‐Mar‐24
Notification state	MG	RS	SP	SP	CE
Sex	Male	Female	Male	Female	Female
Age (years)	11	19	5	21	5
Race	Mixed individual	White	White	White	White
Housing region	Rural	Rural	Urban	Urban	Urban
Nosocomial infection	No	No	No	No	No
Clinical signs and symptoms					
Fever	No	Yes	Yes	No	Yes
Cough	Yes	No	Yes	No	No
Sore throat	No	No	No	No	No
Dyspnea	Yes	Yes	Yes	Yes	No
Respiratory discomfort	No	Yes	Yes	Yes	No
Peripheral oxygen saturation < 95%	Yes	Yes	Yes	Yes	No
Diarrhea	No	No	Yes	No	No
Vomiting	No	Yes	No	No	No
Fatigue	No	Yes	No	No	No
Another symptom^a^	No	Yes	No	No	No
Comorbidities					
Cardiovascular disease	No	Yes	No	No	No
Hematological disorder	No	No	No	No	No
Down syndrome	No	No	No	No	No
Liver disease	No	Yes	No	No	No
Asthma	No	No	No	No	No
Diabetes mellitus	No	Yes	No	No	No
Neurological disorder	No	Yes	No	Yes	No
Chronic lung disease	No	No	No	No	No
Immunosuppression	No	No	No	No	No
Kidney disease	No	No	No	No	Yes
Obesity	No	No	No	No	No
COVID‐19 immunization status	Yes	No	Yes	No	No
Antiviral to treat flu symptoms	No	No	No	No	No
Intensive care unit admission	No	No	Yes	Yes	No
Requirement for ventilatory support	No	Noninvasive	No	Noninvasive	No
Time interval between hospitalization and symptom onset (day)	2	8	2	7	7
Time interval between outcome and symptom onset (day)	8	10	6	10	9
Time interval between outcome and hospitalization (day)	6	2	4	3	3
Length of stay in the intensive care unit (day)	0	0	1	2	0
Outcome	Hospital discharge	Death	Hospital discharge	Hospital discharge	Hospital discharge

Abbreviations: CE, Ceará; MG, Minas Gerais; RS, Rio Grande do Sul; SP, São Paulo.

^a^It is important to note that the category “other symptoms” was not described in detail within the database. In the Open Data of the Brazilian Unified Health System (SUS, of the Portuguese Sistema Único de Saúde) (Open‐Data‐SUS) platform, this variable is recorded in a grouped and nonspecific manner, without individual clinical characterization of the reported manifestations. Therefore, it was not possible to further stratify or accurately describe these additional symptoms in the present study.

With regard to the three patients with Silver–Russell syndrome, two were 5 years old, one male and the other female, and the third was 21 years old and female. All were White and lived in urban areas; two of the three patients had dyspnea, respiratory discomfort, and peripheral oxygen saturation < 95% on hospital admission. With the exception of the 21‐year‐old patient who had neurological disorders and the 5‐year‐old patient who had kidney disease, the patients had no comorbidities of other systems. Only the 5‐year‐old patient had been vaccinated against COVID‐19, and he and the 21‐year‐old patient required hospitalization in an intensive care unit. The 21‐year‐old was the only patient to use noninvasive mechanical ventilation. All the patients were discharged from the hospital (Table 1, Figure 1).

Regarding the time intervals analyzed, symptom onset preceded hospitalization by 2–8 days among patients with Berardinelli–Seip syndrome and by 2–7 days among those with Silver–Russell syndrome. The time between symptom onset and final outcome ranged from 8 to 10 days in Berardinelli–Seip cases and from 6 to 10 days in Silver–Russell cases.

The interval between hospitalization and outcome varied from 2 to 6 days in patients with Berardinelli–Seip syndrome and from 3 to 4 days in those with Silver–Russell syndrome, reflecting relatively short hospital stays overall. Length of stay in the intensive care unit was limited: patients with Berardinelli–Seip syndrome did not require intensive care unit admission, while two patients with Silver–Russell syndrome required brief intensive care unit stays of 1 and 2 days, respectively. Overall, the temporal progression from symptom onset to clinical resolution or death was under 2 weeks in all cases.

**FIGURE 2:**
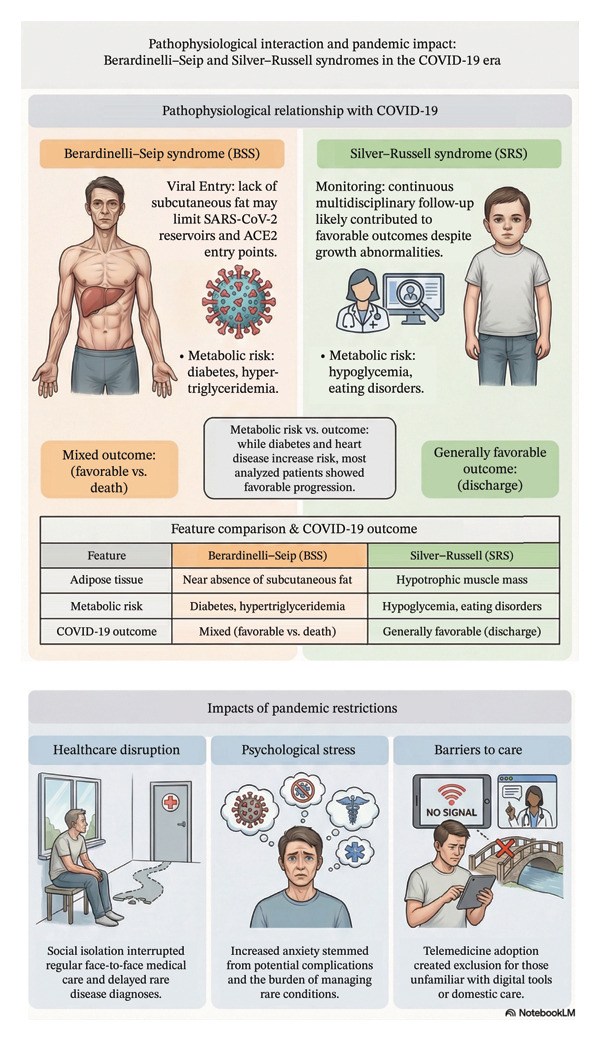
Pathophysiological interaction and pandemic impact in BSS and SRS during coronavirus disease (COVID)‐19. The figure compares key clinical and metabolic features of BSS and SRS relevant to severe acute respiratory syndrome coronavirus 2 (SARS‐CoV‐2) infection, including adipose tissue distribution, metabolic risk, and observed clinical outcomes. While BSS patients may have reduced viral entry points due to lack of subcutaneous fat, metabolic comorbidities remain significant. SRS patients, despite growth and metabolic abnormalities, generally showed favorable outcomes, likely influenced by regular multidisciplinary monitoring. The lower panel illustrates the impact of pandemic restrictions, including healthcare disruption, psychological stress, and barriers to telemedicine among individuals with rare diseases. ACE‐2: angiotensin‐converting enzyme 2.

Figure 2 summarizes the pathophysiological interaction between Berardinelli–Seip syndrome and Silver–Russell syndrome in the context of COVID‐19, as well as the broader impact of pandemic‐related restrictions on individuals with rare diseases. In Berardinelli–Seip syndrome, the near absence of subcutaneous fat may reduce potential SARS‐CoV‐2 reservoirs, although metabolic comorbidities such as diabetes mellitus and hypertriglyceridemia remain important risk factors. In Silver–Russell syndrome, despite growth abnormalities and metabolic vulnerabilities, continuous multidisciplinary follow‐up likely contributed to favorable outcomes. The figure also highlights how social isolation, healthcare disruption, and barriers to telemedicine disproportionately affected patients with rare conditions during the pandemic.

## 3. Discussion

There is only one study in the literature that assessed the impact of the pandemic on people with Berardinelli–Seip syndrome [16]. The cross‐sectional study carried out in Brazil in 2020 included 22 people with congenital generalized lipodystrophy followed up at the Endocrinology Service of the Walter Cantídio University Hospital, the headquarters of the Brazilian Study Group on Hereditary and Acquired Lipodystrophies. Of the 22 patients, only (*N* = 8 [36.4%]) presented reactive results for the serological test against SARS‐CoV‐2. Of these, the majority were female (*N* = 5 [62.5%]). All the patients who tested positive had low high‐density lipoprotein and hypertriglyceridemia and 6 of them had diabetes mellitus; with regard to clinical symptoms, the majority of patients were asymptomatic (*N* = 5 [62.5%]), and, among the reported symptoms, the main ones were fever, diarrhea, and nausea, each of these symptoms affecting 3 (37.5%) patients [16].

Our findings revealed a clinical course that, although marked by the severity that warranted hospitalization, resulted in favorable outcomes in most cases, both contrasting with and expanding upon the data reported by Madeira et al. [16]. While Madeira et al. identified a 0% mortality rate and 62.5% of asymptomatic cases in an outpatient cohort of 22 patients, our study captured a more severe spectrum of disease, with ventilatory support required in 40% of cases and a mortality rate of 20%. This divergence likely reflects ascertainment bias related to hospitalization criteria for severe acute respiratory infection in Brazil, whereas Madeira et al. captured less acute community‐based cases.

Regarding the comorbidity profile and laboratory findings, both studies confirmed the substantial metabolic burden associated with Berardinelli–Seip syndrome. Despite these vulnerabilities, the recovery of 80% of our hospitalized patients supports the “Adipose Tissue Reservoir” hypothesis [16]. The marked reduction in subcutaneous adipose tissue in Berardinelli–Seip syndrome may be associated with lower angiotensin‐converting enzyme (ACE‐2) receptor density and a modified renin–angiotensin–aldosterone system response, suggesting that this phenotypic resilience could mitigate acute inflammatory deterioration, even in the presence of a high‐risk metabolic profile [16].

It is now clear that metabolic disorders such as diabetes mellitus, hypertriglyceridemia, and obesity are associated with more severe forms of COVID‐19 and worse outcomes [26]. SARS‐CoV‐2 enters cells through the binding of its spike glycoprotein with the carboxypeptidase receptor, related to the ACE‐2; obesity, diabetes mellitus, and the proinflammatory state found in people with metabolic disorders increase the expression of SARS‐CoV‐2 entry factors in adipose tissue [26–28]. However, as described in the literature, patients with congenital generalized lipodystrophy have a shortage of subcutaneous adipose tissue, which is a reservoir for SARS‐CoV‐2 binding proteins, and this, together with the fact that most patients are young, may have influenced a better prognosis [16].

To date, no studies have specifically evaluated patients with Silver–Russell syndrome in the context of COVID‐19. However, some prognostic hypotheses can be proposed based on the clinical presentation of the disease. Approximately 84%–100% of patients present with feeding difficulties, frequently accompanied by hypoglycemia, gastrointestinal disorders, and, in some cases, the need for enteral feeding support. In addition, neurodevelopmental delay and skeletal abnormalities may also be observed. Taken together, these factors could suggest a potentially worse prognosis in the event of SARS‐CoV‐2 infection [17]. Nevertheless, all three patients evaluated in the present study were discharged from the hospital without requiring invasive mechanical ventilation.

A possible explanation for this more favorable clinical course may be related to the fact that individuals with Silver–Russell syndrome typically undergo regular multidisciplinary follow‐up aimed at monitoring metabolic parameters and body composition in order to prevent long‐term complications [29]. Furthermore, due to ongoing clinical recommendations and continuous health assessments, these patients often maintain relatively stable health conditions, which may have positively influenced their response to COVID‐19, especially considering that comorbidities such as cardiovascular abnormalities, commonly reported in this population, were not identified in our sample [30].

Moreover, Silver–Russell syndrome is a rare genetic disorder, and specific genetic and immunological characteristics may influence the host response to infectious agents. Although further investigation is required to substantiate this hypothesis, it is possible that such patients exhibit a distinct immune response to SARS‐CoV‐2 infection. Nonetheless, more robust studies are necessary to better elucidate the relationship between COVID‐19 and Silver–Russell syndrome.

Although there appears to be no phenotypic relationship between the severity associated with COVID‐19 and the presence of Berardinelli–Seip and Silver–Russell syndromes in hospitalized patients, it is known that the pandemic has brought challenges to the diagnosis of rare diseases [11]. At the same time, there have been social impacts on rare disease populations during the pandemic, as the already existing difficulties in a “diagnostic odyssey”, with multiple consultations and laboratory tests before a definitive diagnosis, have been aggravated by the pandemic due to social isolation, disruption, and overloaded access to health services and care [31–33]. This disruption to regular face‐to‐face medical care has led to increased anxiety, given the potential risk of serious complications from rare conditions interacting with COVID‐19, which often involves multiple body systems and disables careful management [31, 34]. In addition, the accelerated adoption of telemedicine during the pandemic, although essential for the continuity of some care, created barriers for patients with limited access to digital technologies or who were unfamiliar with such tools, as well as requiring therapeutic adaptations to the domestic setting, further exacerbating the patient’s feeling of social exclusion [31].

Furthermore, despite the findings of this study, there is still the possibility that genetic variability innate to the rare disease is a risk factor for SARS‐CoV‐2 infection. In view of the above, in the literature, it has been reported that, among 2518 genes analyzed, causing 3854 rare diseases, a total of 254 genes have a direct effect on the molecular mechanism of COVID‐19 and 207 have an indirect effect [34]—genetic variants can interact with the molecular mechanisms of SARS‐CoV‐2 infection, affecting crucial immune, inflammatory, and metabolic signaling pathways [34]. These interactions have possibly made differential diagnosis difficult during the pandemic, since COVID‐19 symptoms could mask or exacerbate typical manifestations of rare diseases, such as hematological disorders, immunodeficiencies, and neuropathies [34]. In this context, mechanistic modeling tools have emerged as important allies, allowing us to identify specific molecular interactions and broaden our understanding of the relationship between these rare conditions and COVID‐19 [35]. In addition to optimizing diagnosis, these approaches have opened the way for personalized approaches, including targeted clinical monitoring and treatment adjustment, ensuring safer and more effective care for rare patients.

The novelty of this study resides in its nationwide, population‐based assessment of hospitalized patients with two rare genetic syndromes, Berardinelli–Seip and Silver–Russell, over four years of the COVID‐19 pandemic in Brazil. While COVID‐19 outcomes have previously been described in small cohorts of patients with Berardinelli–Seip syndrome, there are, to our knowledge, no prior reports specifically evaluating hospitalized individuals with Silver–Russell syndrome in this context. Moreover, the simultaneous analysis of both conditions using a large national public health database provides a broader epidemiological perspective and contributes original evidence to the limited literature addressing the interaction between rare genetic diseases and COVID‐19.

It is important to note that the database used in this study comprises around two million patients hospitalized due to severe acute respiratory infection caused by SARS‐CoV‐2 and therefore represents a broad sample of the Brazilian population; however, caution should be exercised when generalizing the findings. The researchers do not have access to individual medical records, detailed clinical histories, physical examination findings (e.g., respiratory rate, blood pressure, temperature, and body mass index), imaging studies (e.g., chest X‐ray or computed tomography), comprehensive laboratory parameters (including lipid profile, triglycerides, inflammatory markers, or full blood chemistry), specific treatment regimens, or individualized therapeutic plans, as these variables are not available in the Open‐Data‐SUS hospitalization database.

In addition, the diagnosis of Berardinelli–Seip and Silver–Russell syndromes could not be molecularly confirmed within the dataset, as genetic testing results are not provided in the public platform. Furthermore, the data entered into the system are recorded by healthcare professionals during routine care and are therefore subject to potential misclassification, incomplete reporting, and under‐reporting of comorbidities and clinical variables. It is also important to emphasize that only comorbidities explicitly registered in the database could be analyzed, which may underestimate the true burden of associated conditions.

However, despite these inherent limitations related to secondary surveillance data, this study represents the first nationwide, population‐based description evaluating both Berardinelli–Seip and Silver–Russell syndromes concomitantly in the context of COVID‐19 hospitalization over four years of the pandemic. Finally, contrary to initial expectations, we observed that patients with Berardinelli–Seip and Silver–Russell syndromes generally had favorable outcomes, despite presenting risk factors potentially associated with worse prognosis.

Further investigations with larger cohorts, access to primary clinical data, detailed laboratory and imaging findings, and molecular confirmation of rare disease diagnoses are needed to better elucidate the pathophysiological interactions between SARS‐CoV‐2 infection and rare genetic syndromes. Such studies may contribute to improving risk stratification, optimizing clinical management, and advancing knowledge regarding the intersection between rare diseases and emerging infectious diseases. In addition, multinational collaborative initiatives conducted during the pandemic, such as those supported by the National Institute for Health and Care Research (NIHR), may further enhance understanding of rare diseases by enabling harmonized data collection, broader population representation, and more robust comparative analyses across different healthcare systems [35–39].

## 4. Conclusion

In summary, the analyzed data indicate that patients with Berardinelli–Seip and Silver–Russell syndromes hospitalized for COVID‐19 in Brazil generally showed favorable clinical outcomes, despite metabolic and systemic features that could theoretically worsen prognosis. The single unfavorable outcome was associated with a higher burden of comorbidities and lack of vaccination, reinforcing the importance of immunization and continuous clinical management in these individuals. Although limited by the small number of cases and the use of a secondary database, these findings help reduce the knowledge gap regarding COVID‐19 in rare diseases and support the need for collaborative studies with larger cohorts and integrated clinical–molecular data to better guide risk stratification and personalized care for these populations.

## Author Contributions

Luiz Felipe Azevedo Marques and Fernando Augusto Lima Marson collected and tabulated the data. Luiz Felipe Azevedo Marques, Adriele Evelyn Ferreira Silva, Patrícia Teixeira Costa, Lucas Silva Mello, Vinícius Santiago dos Santos, and Fernando Augusto Lima Marson interpreted the study findings. Luiz Felipe Azevedo Marques, Adriele Evelyn Ferreira Silva, Patrícia Teixeira Costa, Lucas Silva Mello, Vinícius Santiago dos Santos, and Fernando Augusto Lima Marson wrote and revised the text thoroughly before submitting the manuscript to the scientific journal. Luiz Felipe Azevedo Marques, Adriele Evelyn Ferreira Silva, Patrícia Teixeira Costa, Lucas Silva Mello, Vinícius Santiago dos Santos, and Fernando Augusto Lima Marson approved the manuscript and agreed with its submission to the scientific journal.

## Funding

Luiz Felipe Azevedo Marques received a grant from the São Paulo Research Foundation (FAPESP, of the Portuguese Fundação de Amparo à Pesquisa do Estado de São Paulo), for the development of the undergraduate research, grant no. 2024/20061‐9. Adriele Evelyn Ferreira Silva received a grant from the FAPESP for the development of master’s thesis, grant no. 2024/13359‐1. Lucas Silva Mello received a grant from the National Council for Scientific and Technological Development (CNPq, of the Portuguese Conselho Nacional de Desenvolvimento Científico e Tecnológico) for the development of the undergraduate research, grant no 8887.823904/2023‐00. Vinícius Santiago dos Santos received a grant from the FAPESP for the development of the undergraduate research, grant no. 2024/20055‐9. Fernando Augusto Lima Marson acknowledges support from the CNPq, Brazil, through a Research Productivity grant (no. 305906/2024‐0).

## Disclosure

Prior Presentation and Preprint: This manuscript has not been previously published, is not available as a preprint, and has not been presented at any scientific conference or seminar.

## Ethics Statement

The study was conducted in accordance with the Declaration of Helsinki and was approved by the Institutional Research Ethics Committee (Certificate of Presentation for Ethical Appreciation no 67241323.0.0000.5514; Approval no 5.908.611). No written consent has been obtained from the patients as there is no patient identifiable data included in this case report.

## Conflicts of Interest

The authors declare no conflicts of interest.

## Data Availability

The data used in this study are publicly available from Open Data of the Brazilian Unified Health System (SUS, of the Portuguese Sistema Único de Saúde) (Open‐Data‐SUS), the official open‐access platform maintained by the Brazilian Ministry of Health, which provides anonymized public health datasets. The curated dataset and analytical procedures applied in this study are available from the corresponding author upon reasonable request.
